# Convolutional Neural Network Based Real Time Arabic Speech Recognition to Arabic Braille for Hearing and Visually Impaired

**DOI:** 10.3389/fpubh.2022.898355

**Published:** 2022-05-27

**Authors:** Surbhi Bhatia, Ajantha Devi, Razan Ibrahim Alsuwailem, Arwa Mashat

**Affiliations:** ^1^Department of Information Systems, College of Computer Sciences and Information Technology, King Faisal University, Al Hasa, Saudi Arabia; ^2^Research Head, AP3 Solutions, Chennai, India; ^3^Faculty of Computing and Information Technology, King Abdulaziz University, Rabigh, Saudi Arabia

**Keywords:** braille, Speech to Text, speech to Braille, speech recognition, text to braille, Arabic digit

## Abstract

Natural Language Processing (NLP) is a group of theoretically inspired computer structures for analyzing and modeling clearly going on texts at one or extra degrees of linguistic evaluation to acquire human-like language processing for quite a few activities and applications. Hearing and visually impaired people are unable to see entirely or have very low vision, as well as being unable to hear completely or having a hard time hearing. It is difficult to get information since both hearing and vision, which are crucial organs for receiving information, are harmed. Hearing and visually impaired people are considered to have a substantial information deficit, as opposed to people who just have one handicap, such as blindness or deafness. Visually and hearing-impaired people who are unable to communicate with the outside world may experience emotional loneliness, which can lead to stress and, in extreme cases, serious mental illness. As a result, overcoming information handicap is a critical issue for visually and hearing-impaired people who want to live active, independent lives in society. The major objective of this study is to recognize Arabic speech in real time and convert it to Arabic text using Convolutional Neural Network-based algorithms before saving it to an SD card. The Arabic text is then translated into Arabic Braille characters, which are then used to control the Braille pattern via a Braille display with a solenoid drive. The Braille lettering triggered on the finger was deciphered by visually and hearing challenged participants who were proficient in Braille reading. The CNN, in combination with the ReLU model learning parameters, is fine-tuned for optimization, resulting in a model training accuracy of 90%. The tuned parameters model's testing results show that adding the ReLU activation function to the CNN model improves recognition accuracy by 84 % when speaking Arabic digits.

## Introduction

Louis Braille invented Braille, a worldwide method of reading and writing for visually impaired people, in 1821. The Braille cell is the system's smallest unit. Because of the prosody (intonation) of the system, visually and hearing-impaired individuals who are skilled in Finger Braille can communicate words and express a variety of emotions (1). Because there are so few people who can read Finger Braille, visually and hearing-impaired people can only communicate through an interpreter. As a result, those who are visually and hearing impaired are severely limited in their participation. Finger Braille input techniques, including a wearable device, have been developed (2). Created a wearable input device employing accelerometers affixed on the tops of rings and invented finger Braille gloves with accelerometers integrated in the fingers (3). In addition (4), created a wristband with an eighteen-accelerometer Finger Braille input device.

Visually and hearing-impaired people must wear gloves, rings, or bracelets to input Finger Braille (5) into these devices. People who use these assistive devices (6) who are blind and deaf must not only wear the sensors, but also learn a new communication system. Children who have both hearing and vision impairments require different teaching strategies than children who only have one impairment. Although different countries define hearing and vision impairments differently, the negative effects of dual sensory impairments on access to environmental information are universally acknowledged, as is the fact that this unique disability necessitates specific teaching strategies to aid and support learning.

Because they cannot read text written on regular paper, visually impaired people use the Braille system in their writing and reading. However, Visual and hearing individuals in UAE are deprived of education and information due to a lack of basic reading materials, such as books and notes in Braille written format. As a result, we created an automatic Arabic speech to Braille translation system to address this issue by converting electronic Arabic books to Arabic Braille books.

### Braille System

The Braille system (7) is a reading and writing system used worldwide by visually impaired people. Passing fingers across Braille characters or Braille cells is how it's read. In a Braille cell, six dots are arranged in a rectangle grid of two dots horizontally in a row and three dots vertically in a column, yielding four different patterns. Each dot arrangement is known as a cell (1), and each cell will have at least one elevated dot and a maximum of six.

**Figure 1:**
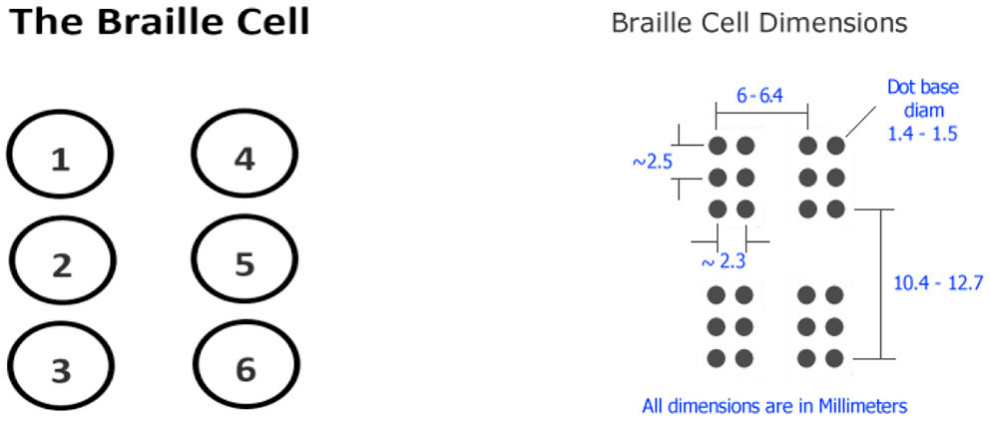
Braille Cell.

Each of the twenty-five rows of text on a standard Braille printed page has forty cells. The size of a standard Braille page is about 11 inches by 11 inches. A Braille cell's size is also specified, though it varies greatly depending on the country. The dimensions of a Braille cell as printed on an embosser are shown in Figure 1.

The inventor of the Braille system anticipated the need to convey information in a compact format so that a group of cells could convey a lot more information in the string of letters that make up the cells. The cell's six dots (7) can be combined to make 63 different dot patterns. Despite the fact that there are a total of 64 patterns, the final one is a cell with no dots, which serves as a gap. When viewed through the lens of text representation in a computer, a Braille cell is thus analogous to a 6-bit character code. Almost all information can now be created electronically, and computer use in daily activities (typing and printing) has increased significantly. As a result, more specialized technologies that are accessible to visually impaired individuals are critical, allowing them to use computers for everyday transactions and contribute as much to society and the evolution of IT as others.

### Automatic Arabic Speech Recognition—AASR

**Figure 2:**
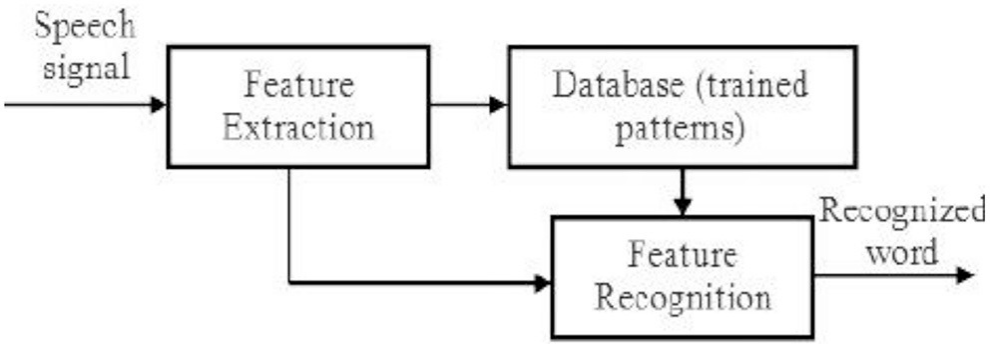
Automatic Arabic speech recognition AASR.

Automatic Arabic Speech Recognition (AASR) (8) is a system that converts human speech into text. It's a difficult task because human speech signals are highly unpredictable due to a variety of speaker characteristics, speaking styles, and imprecise background noises (9). Variable-length voice signals must also be translated into variable-length word or label sequences by AASR. An AASR system is made up of many components, as shown in Figure 2. A microphone captures the speech signal first and foremost, which is then saved in digital format (10). The voice signal is non-stationary and fluctuates a great deal. As a result, the signal is divided into small chunks known as frames, with each frame's signal becoming more stationary and its features represented by a fixed length feature vector (11, 12). The procedure is referred to as feature extraction (13, 14), and the component that performs it is referred to as the front end. The front end's output is the observation sequence X, with each observation being a feature vector representing a single frame.

The acoustic model's goal is to simulate the acoustic characteristics of various speech units, as well as the relationship between the acoustic aspects of the entire voice utterance and the utterance label sequence or text. For spoken acoustic signals, the hidden Markov model (HMM) (15) is a popular and successful model. The HMM is very versatile when it comes to modeling variable length sequences, and it tolerates changes in speaking speeds.

It models the likelihood of getting the speech observation sequence given a set of latent (hidden) discrete states. A decoder is used to recognize speech based on the acoustic and language models to model the relationship between an HMM state and the speech observations that pertain to this state. The decoder component searches for the best label sequence for a given voice utterance that maximizes the scores computed by the acoustic (16) and linguistic models. Because the number of viable label sequences grows exponentially with the length of the input feature sequence, the efficiency and speed of the decoder are critical elements of a successful ASR system (17).

#### Feature Extraction on ASR

The initial stage in speech recognition is the extraction (13, 14) of a sequence of feature vectors X that reflects the input voice signal. Feature vectors (18) are available in a wide range of sizes and shapes. In an acoustic model-based system, a good feature vector should include all relevant information for classification of the input signal (19). It's also a good idea to get rid of any data that isn't absolutely necessary. Mel frequency spectral coefficients (MFSC) and Mel-frequency cepstral coefficients (MFCC) are two feature vectors commonly used in academic and commercial ASR systems (20).

The first step is to take short overlapping windows of the speech signal, making it quasi-stationary and incorporating properties unique to single speech units. A Hamming window (or similar bell-shaped functions) is multiplied by this speech window to reduce the effect of discontinuity at the window's two edges. Because distinct speech units and sounds differ primarily in the distribution of energy along different frequencies, it is common practice to convert the speech time domain data into a frequency domain signal using Fast Fourier Transform (FFT) analysis (21). Filter bank analysis is then used to measure the energy in a small number of frequency ranges. These filters are dispersed along the Mel scale frequency to imitate human ear's sensitivity to frequency variances (22). Mel frequency filter bank analysis employs triangular filters. The energy in each filter is estimated to estimate the MFSC feature.

In practice, the log is used to calculate log-MFSC characteristics, which are closer to the sensitivity of the human ear and allow for better discrimination. In fact, neural network models, particularly convolutional neural networks, benefit from the log-MFSC feature. The log-MFSC features are closely correlated because the spectral envelop changes smoothly with frequency and neighboring filters overlap. The discrete cosine transform is used to minimize correlation in order to generate MFCC features (23). When using Gaussian Mixture Models (GMMs), this is especially important when using a diagonal covariance matrix.

#### HMM Based ASR System

An HMM can be used in a variety of ways to recognize speech. Isolated speech recognition is the most basic type, in which the system's goal is to classify individual words. In this scenario, each word is modeled using a different HMM model. The number of distinct acoustic portions of the word corresponds to the number of states in each HMM model. To classify the segment, the term associated with the model with the highest likelihood is used. The likelihood is calculated either exactly using the forward probability or roughly using the Viterbi algorithm's likelihood of the optimum state sequence in this scenario.

Speech recognition that is connected (continuous) is a more advanced technology that allows the speaker to continuously utter a large number of words. In this scenario, different word models are combined to create a large composite HMM. The types of transitions that are allowed between words are defined by a grammar. Furthermore, a language model is frequently used to assign different weights to different word transitions. Speech recognition is accomplished by determining the most likely state sequence and mapping that sequence into a word sequence that represents the uttered sentence. The disadvantage of this model is that learning a good model for each word necessitates a large amount of training data. Furthermore, the model is incapable of recognizing terms not found in the training data.

To overcome these difficulties, models that represent sub-word units such as phonemes can be used. In these models, each phoneme is represented by a three-state sequence that includes the phoneme's transitional beginning and end, as well as the middle stationary state. To represent a word, a series of phoneme models are combined to form a word model. A dictionary is used to specify the phoneme models that represent a word. This method has the advantage of sharing each phoneme's training data across multiple words, reducing the amount of data needed for training. It can also recognize unseen words if they have equivalent entries in the dictionary.

#### Neural Network (NN) Based ASR

As a result of improved performance on a variety of speech recognition tasks, deep neural network models (DNNs) have recently resurfaced (26). Previous attempts to use NNs in acoustic modeling attempted to use the NN as a separate model for detecting simple speech units. To replicate the Viterbi algorithm's work in Lippmann (27), devised a recurrent neural architecture dubbed Viterbi net. As a result, it can recognize speech almost instantly. However, it lacked a training algorithm. The weights of the Recurrent Neural Network (RNN) were calculated using a trained HMM model. As a neural network architecture, the Time Delay Neural Network (TDNN) was proposed in Waibel et al. (28). The TDNN combines longer input contexts by using a series of consecutive input frames and hidden activations in the top layers. The model was used to classify a small number of phonemes. To successfully use NNs for ASR, a hybrid NN-HMM model was used. In one version of the model that has recently gained popularity, the NN replaces the GMM in scoring speech frames, while the HMM is used to model the speech signal's sequential temporal behavior.

## Research Methodology

The Arabic language is the world's largest and most experienced Semitic language (26), and it differs from other European dialects such as English in several ways. Arabic is the Arab world's official language. Arabic is the world's sixth most communicated language, based on the number of people who speak it as their first language. Furthermore, ~250 million people speak Arabic as their first language. Arabic sentences are written from right to left, and some letters may change shape depending on their position within a word (29).

Speech to-Braille STB (30) is a branch of computer science that focuses on developing computer frameworks that can recognize expressed words from an amplifier and convert them to messages. Speech to Text is the process of converting or deciphering an audible signal obtained by a receiver or a phone into a string of words. Extraction of highlights from a gathered signal and matching them to a voice test in an information base is used to perceive a word (31, 32).

The goal of the procedure is to create a Digit speech recognition refers to the recognition of utterances in which the user can speak normally without pausing between words, which is similar to how humans speak. Subword sounds, such as phonemes (alphabets in Indian languages) (29), are commonly used as the basic unit of recognition in continuous speech recognition systems. The sequence of phonetic models forms the larger linguistic unit, such as words/sentences, during recognition. Because the number of sub-word units is limited, collecting enough data to train each of these basic sounds in a variety of acoustic environments is not difficult. Adding a new word is simple because all that is required is a description of the new word in terms of previously learned speech sounds. Among the features are speech analysis, training tools, recognition tools, outcome analysis, and labeling tools. It can recognize both single and related words. It is capable of modeling both full words and sub-word units.

**Figure 3:**
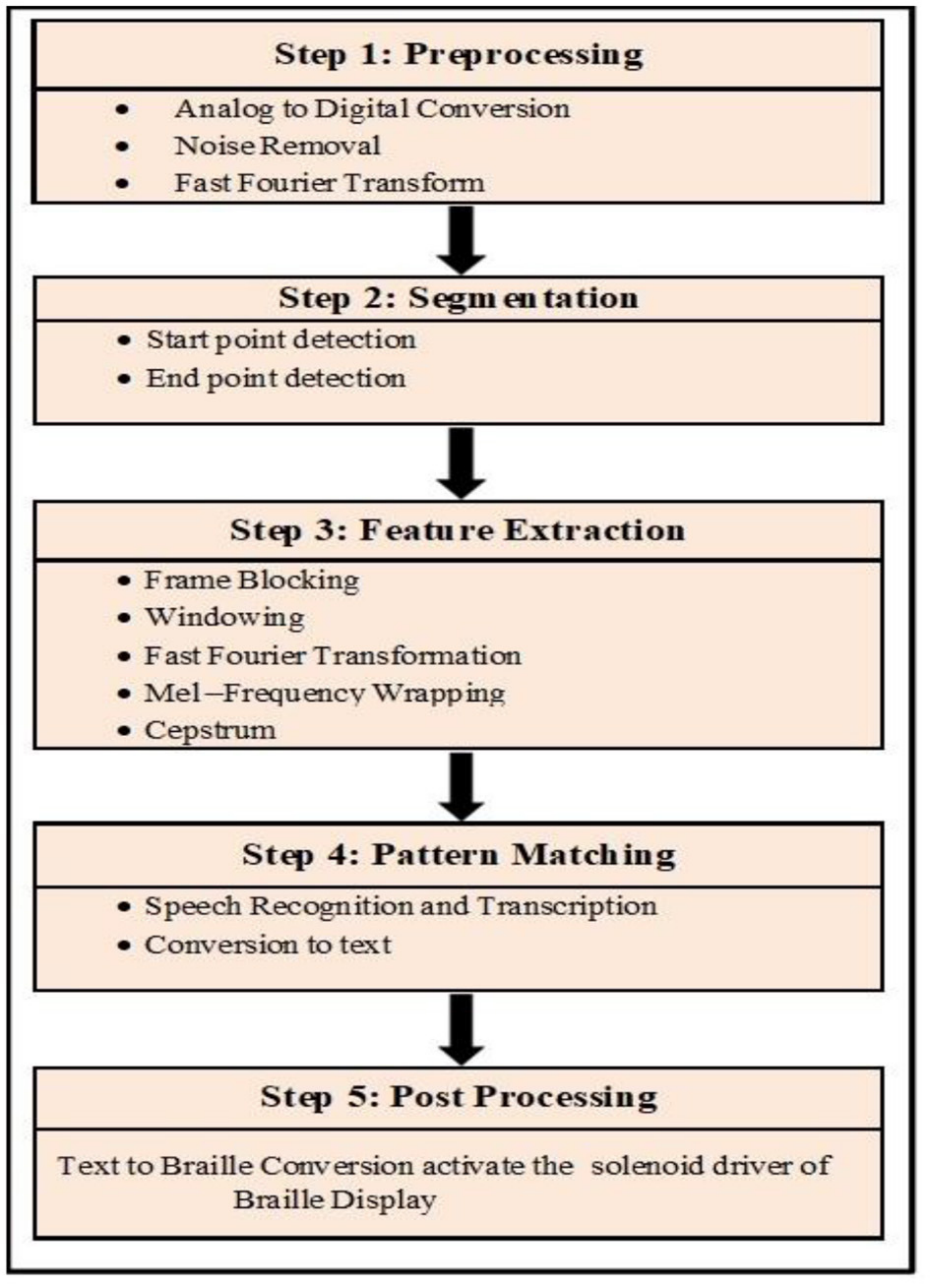
Flow of Proposed Methodology.

The system's post-processing involves retrieving the exact data associated with the recognized speech. As shown in Figure 3, the fetched Arabic data corresponding to the Arabic Corpus dataset is converted into Arabic Braille script and delivered to the user in the form of Braille Display.

## Convolutional Neural Networks for ASR

Speech signals can take many different forms that have little to do with the speech's grammatical content. For example, different speakers may have different voice qualities. There can be a lot of differences between speakers, even if they are the same person. Variations in speaking speed, pitch, and formant frequencies due to changes in vocal tract length, as well as background noise, are examples.

A variety of strategies have been proposed to deal with these differences. In this case, there are two approaches: passive and active. The goal of passive strategies is to standardize speech variances. This can be accomplished by employing special features such as MFCCs, which filter out information that is unrelated to the speech's linguistic content. The HMM normalizes rate variations because it is tolerant of variations in speaking pace.

Active approaches aim to estimate some parameters that transform the speech signal or to fine-tune the trained speech models to perform better in new situations. Some speaker adaptation strategies, for example, change the HMM model parameters to reflect the new speaker's characteristics. Other speaker adaptation strategies convert a new speaker's speech signal into a canonical speaker that the acoustic model can learn from. The new model or transformation parameters are calculated from some speech adaption data collected from the new speaker in both situations before performing speech recognition on this speaker.

The Convolutional Neural Network (33) (CNN) is a type of artificial neural network that processes data in the form of patterns. CNN is an effective method for building deep learning models that have been used in object recognition applications. CNN (34) was proposed as a method of reducing the amount of data required for traditional artificial neural networks. Three layers make up a traditional Convolutional Neural Network: convolution, pooling, and fully connected layers (35). The pooling layer is used to reduce the number of values in feature maps, while the convolution layer is used to extract features. The best feature maps are passed into the classifier using a fully linked layer (35, 36). CNN is scalable in general and the training procedure takes less time.

The main advantage of the CNN is that domain knowledge can be used to create the CNN structure in order to manage application-specific variances. In these applications, CNNs are used to take advantage of translation invariance and to tolerate small changes in visual patterns in multiple directions. Weight sharing, local connectedness, and pooling are some of the network topologies used by CNNs, as opposed to standard NNs. As a result, minor image deformations and noise, as well as small rotations and scaling, are less noticeable to the CNN. These invariances are difficult to learn automatically in typical NNs. In a similar way, CNN ideas have recently been applied to voice processing.

Unsupervised learning of acoustic features was achieved using the CNN structure (37). Convolution over time is used in these studies to manage small temporal shifts for better speaker, gender, and phonetic classification representations, but not for audio recognition.

The CNN structure allows us to acquire frequency invariant features when applied along the frequency axis (38, 39). A CNN can also accommodate temporal changes and thus better handle speaking speed variances when applied along the time axis (40). However, the HMM can handle these variances inside the hybrid NN-HMM model, reducing the benefit of applying convolution and pooling over time.

### Description of Arabic Digits

**Figure 4:**
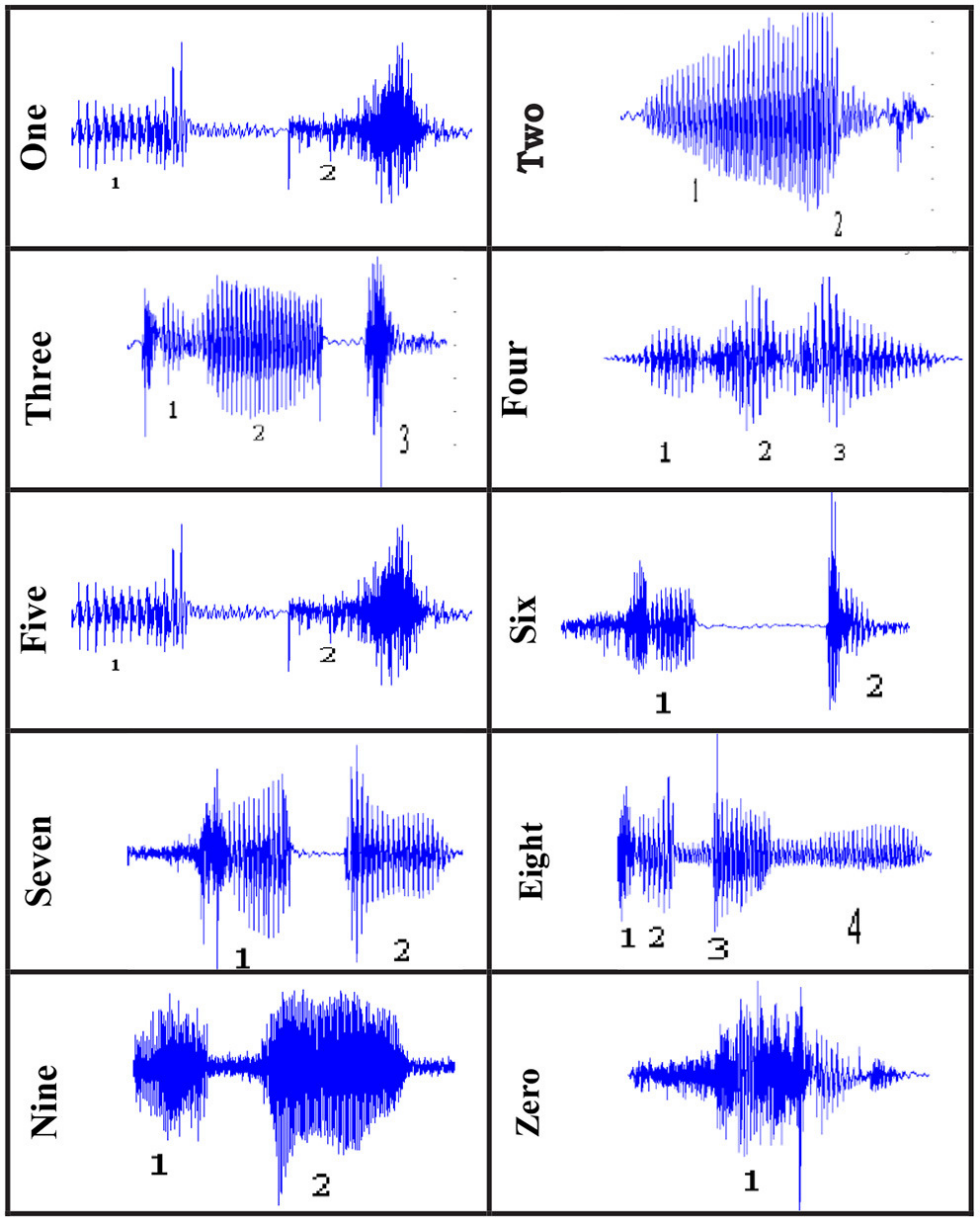
Wave of Arabic Digit.

Due to background noise interference, speech is harmed in voice applications. As a result, we can't tell whether a signal contains valid data just by looking at it (41). Many efforts have been made in speaker system independent, automatic Arabic speech recognition for an isolated digit or continuous digit speech for many languages. There is still a scarcity of research on speech recognition in the Arabic language. All speech recognition applications, including those described here, are, however, available in English. Despite the fact that our study focused on Arabic speech recognition in digit contexts (Figure 4).

### Feature Extraction and Classification Using CNN

**Figure 5:**
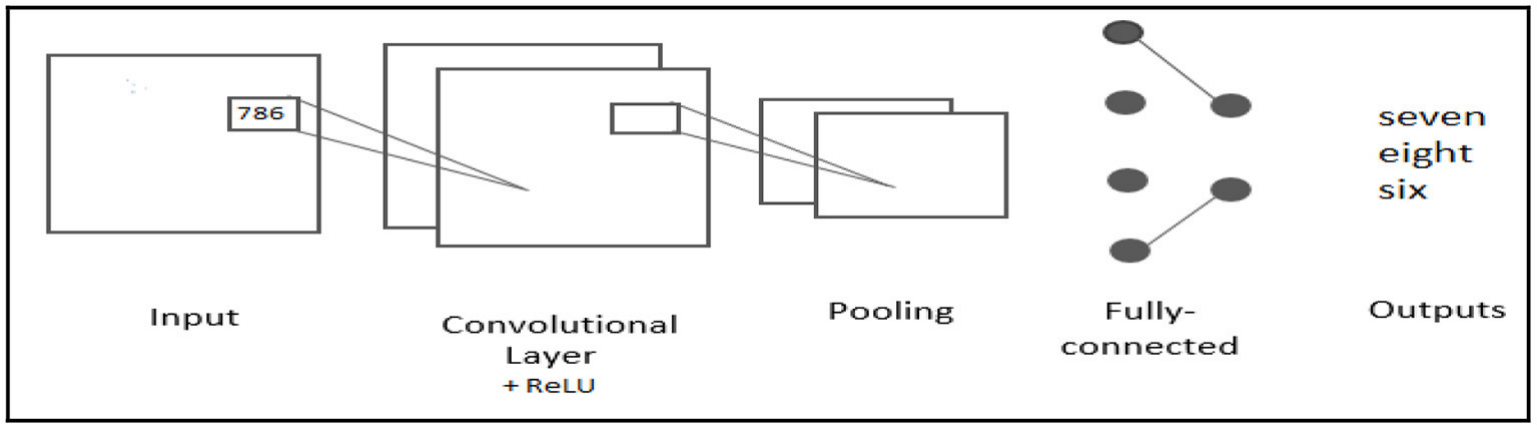
CNN Architecture of Spoken Arabic digit to Text.

The feature extraction technique for audio data is depicted in Figure 5 (42–46). It shows the spectrum of audio data before and after feature extraction using the Python'sklearn' module. The data was split 80:20, with 80% going into the training dataset and 20% into the test dataset, with 20% of the training dataset being used as a validation set for this Speech recognition processing (47). The model processes the training, test, and validation datasets, which will be used in the trained model's test dataset prediction analysis.

- In the convolution layers, we created two separate models, each with its own set of parameters.
- In both models, the first 2D-convolutional layer (conv2D) was used to analyze train data, and it has a 128-bit resolution (Model-1).
- A RELU activation function with a filter size of 64 (model-2).
- The output of the first Conv2D layer was sent to the first batch normalization layer.
- For each model, this procedure is repeated twice using the second and third conv2D.
- The first model employs 48 filters with a normalization pool size of (3, 3), whereas the second employs 32 filters with the remaining two parameters unchanged.

After the feature learning stage, the classification technique starts by flattening the resulting vector into a one-dimensional vector. The data was flattened because categorization is easier with a one-dimensional vector. The 1D vector of the flatten layer is then passed through a series of layers.

Finally, the output vector is sent to a 20-unit dense layer, which corresponds to the number of key words in the Arabic dataset. After that, the final result was obtained. The optimizer in this model is ‘Adam,' and the loss function is ‘Sparse-Categorical Cross-entropy.' Figure 5 depicts the basic architecture of our proposed model.

## Text to Braille

**Figure 6:**
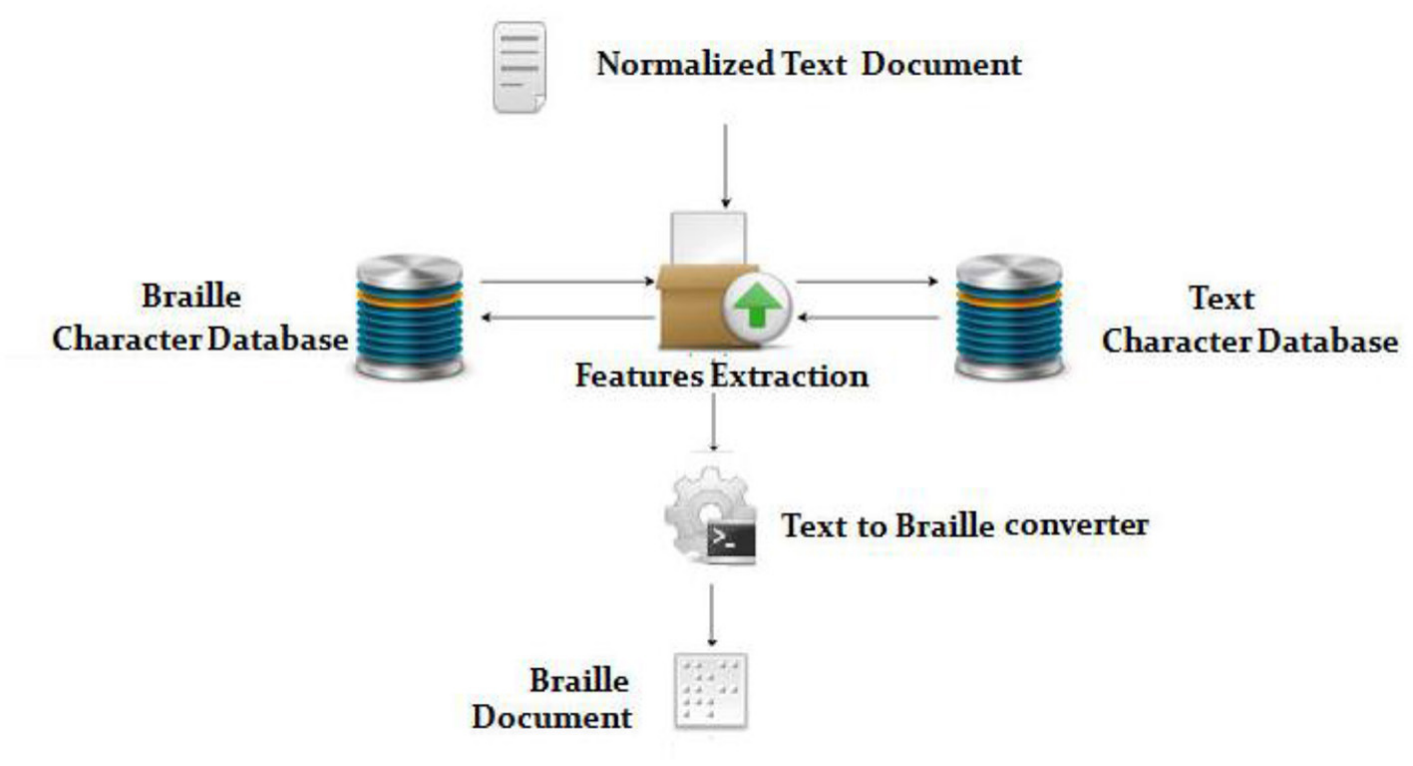
Text to Braille Conversion.

Figure 6 depicts the steps involved in converting text to braille.

- Enter Arabic Text: Entering Arabic text in the provided area or by browsing and attaching a text file (48).
- Validation and data verification of Arabic text: The primary function of this block is to validate and verify the Arabic content extracted from the speech. Validating a meaningful text and verifying the converted word in a database (Text Database) (48) is part of the validation procedure.
- Braille conversion process: The Braille Process converts each character into an equivalent Braille character. The symbols for each and every Arabic digit character can be found in the currently available database (Braille Database) (49–51).
- Arabic Braille Output: After the conversion has been replaced appropriately, the final output, as shown in Figure 6, can be handled through the output interface to reach the end user.

## Experimental Analysis

The Methodology of Speech recognition and Conversion to Braille system (30) has following steps.

**Figure 7:**
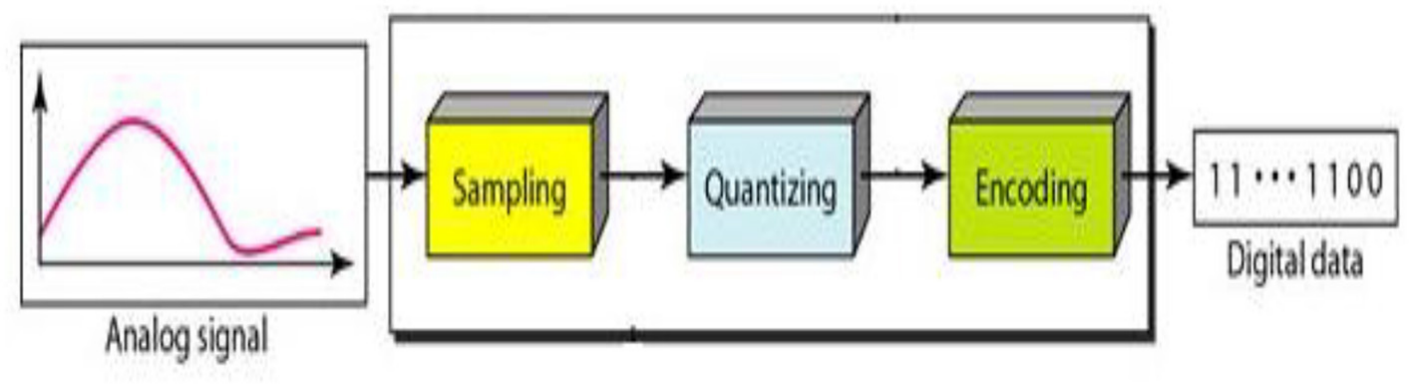
Speech signal Capture.

Step 1: The first step in this research is to gather information from a variety of Arabic speakers. Speakers must record the Arabic numerals 0 to 9 and say each Arabic number separately in a clear voice with minimal background noise, as shown in Figure 7. As a result, data collection was required in order to develop the speech recognition system; this data was collected by various countries around the world, each with its own dialect for pronouncing words (24, 25). Geographical areas influence how speech is delivered, but they aren't always the source of variation; for example, dialects may be influenced by family history. The voice signal is obtained by recording or capturing the entire audible frequency range of a human voice. The sampling frequency is defined as the number of samples obtained in a second.

**Figure 8:**
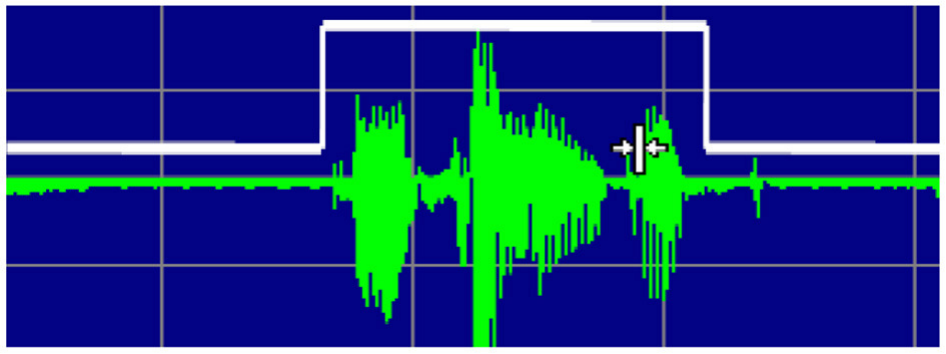
Endpoint Identification.

Step 2: To define where a single utterance begins and ends, the zero-crossing rate (ZCR) and average amplitude thresholds are used. It can be classified as a speech signal segment if the energy in the frame exceeds the energy threshold. However, because unvoiced speech signals are sometimes merged or combined with ambient noises, the marking of start and stop locations may not be precise. Unvoiced speech has a higher ZCR than noise, so it can be used to distinguish between noise and a weak speech signal, allowing for better start and stop point marking, as shown in Figure 8.

Step 3: Using CNN to recognize speech: The classification stage is followed by a fully connected layer and a softmax layer, and the convolutional layers and pooling layers are used to learn features. Except for the fully-connected layer, all convolutional and pooling layers used the ReLU activation function. To extract features, have used the following settings: 16,000 sample rate, 512 and 160 sample FFT lengths and hop sizes, as well as the Hamming window function The first CNN layer conducts a convolutional over the input feature vector using 32 ReLU kernels with 3 × 3 receptive fields and a stride of 1 in both dimensions, as shown in Table 1.

**Table 1:** Performance Table.

**Digit**	**Correct recognition**	**Wrong recognition**	**Accuracy of MFCC**	**Correct recognition (CNN and ReLU)**	**Accuracy After CNN and ReLU**
1	15	5	75%	18	90%
2	13	7	65%	16	80%
3	13	7	65%	16	80%
4	15	5	75%	18	90%
5	13	7	65%	16	80%
6	12	8	60%	15	75%
7	14	6	70%	17	85%
8	13	7	65%	16	80%
9	14	6	70%	17	85%
0	16	4	80%	19	95%
Accuracy % (24)			69%	Accuracy % after CNN & ReLU	84%

**Figure 9:**
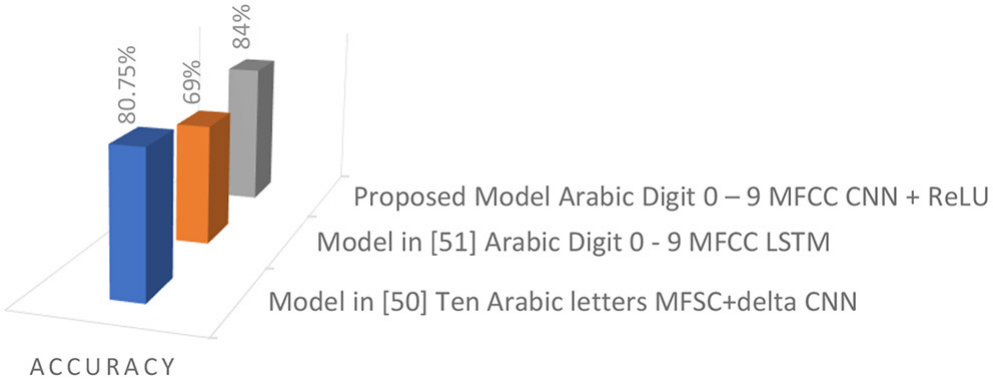
Comparison of Accuracy.

The proposed model was tested using an Arabic speech corpus of isolated words. The used corpus is subjected to a variety of modifications, including pitch, speed, dynamic range, noise, and time shifting forward and backward. As shown in Table 2 and Figure 9, the maximum accuracy achieved when using the ReLU activation function with CNN was 84%. The ReLU activation function, a non-linear activation function, has gained prominence in this proposed methodology. When comparing the results of this study to previous research, it is clear that the proposed methodology performed better in the AASR.

**Table 2:** Comparison of Accuracy.

**Models**	**Dataset**	**Features**	**Recognizer**	**Accuracy**
Model in Rajagede et al. (25)	Ten Arabic letters	MFSC+delta	CNN	80.75%
Model in Wazir and Chuah (24)	Arabic Digit 0–9	MFCC	LSTM	69%
Proposed Model	Arabic Digit 0–9	MFCC	CNN +ReLU	84%

**Figure 10:**
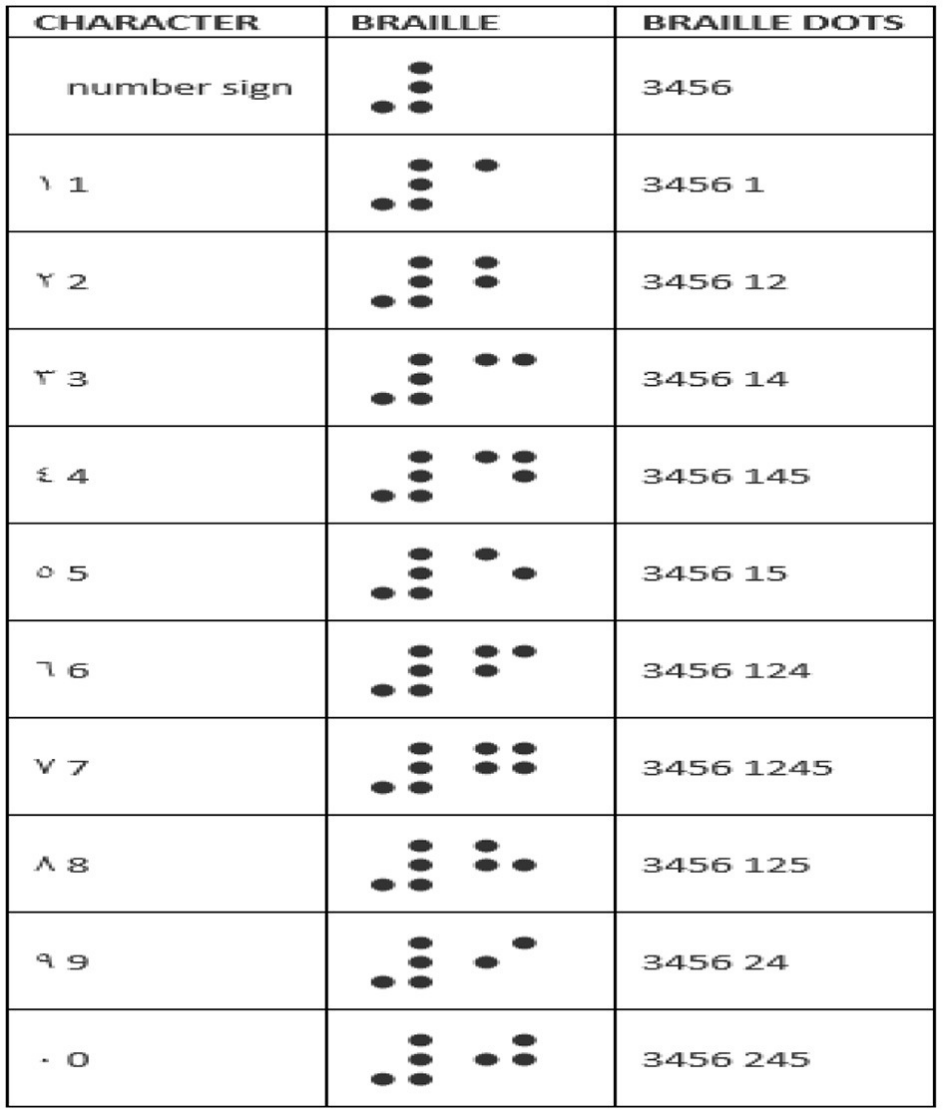
Arabic Digit to Braille.

Step 4: Text to Braille Conversion: The result, as shown in Figure 10, will be processed through the Arabic Text database, which will identify the Arabic text.

**Figure 11:**
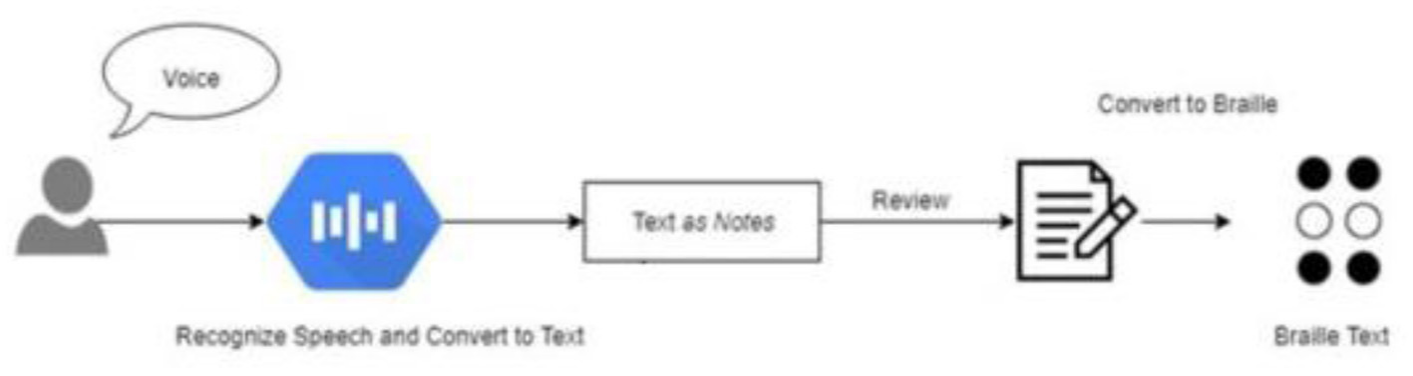
Spoken Arabic Digit to Braille.

Step 5: Postprocessing: The Arabic letter is embossed as Arabic Braille script on the Braille display using solenoid drive (52) as shown in Figure 11. The evaluation is based on how it is handled and prioritized including some other performance parameters. The primary reason for employing the device would be decided by determining if the device's benefit outweighs the negative encountered. Following the prototype's implementation, it is evaluated with a small group of visually challenged people, as well as people who are both visually and hearing impaired.

## Conclusions

Braille is a text-based communication system that is solely dependent on touch. People with vision and hearing impairments would use it. We propose a Speech to Braille conversion procedure to provide the visually and hearing-impaired people can easily make notes in Braille using the provided methods (53) by simply speaking or playing a tape. This app will help people with both visual and hearing impairments communicate in real time, and it will be a cost-effective way. This research produces output that is comparable to traditional high-cost braille printers, as well as assisting visually and hearing-impaired people in recognizing the Speech and converting into Braille output. Speech recognition is a bonus feature that allows visually and hearing-impaired people to print braille for personal use without a computer or a mediator by simply speaking the phrases to be printed into a microphone.

Arabic digits six are particularly difficult to recognize when spoken. The complexity of the voiced signals of the spoken digits is the reason. Deep learning requires a large amount of data to achieve better accuracy, the accuracy of CNN model could be greatly improved by using a large amount of training data. More digit voice samples with noise, for example, could give us a more robust classifier. It's worth noting that as the amount of training data grows, so does the amount of time spent training.

## Data Availability Statement

The original contributions presented in the study are included in the article/supplementary material, further inquiries can be directed to the corresponding author/s.

## Author Contributions

AD and SB: conceptualization, investigation, and methodology. SB and RA: funding acquisition and resources. AM: project administration and visualization. AD: writing of the original draft. AM and SB: writing of the review and editing. SB: validation. RA: data curation. All authors contributed to the article and approved the submitted version.

## Funding

This work was supported through the Annual Funding track by the Deanship of Scientific Research, Vice Presidency for Graduate Studies and Scientific Research, King Faisal University, Saudi Arabia (Project No. AN000602).

## Conflict of Interest

The authors declare that the research was conducted in the absence of any commercial or financial relationships that could be construed as a potential conflict of interest.

## Publisher's Note

All claims expressed in this article are solely those of the authors and do not necessarily represent those of their affiliated organizations, or those of the publisher, the editors and the reviewers. Any product that may be evaluated in this article, or claim that may be made by its manufacturer, is not guaranteed or endorsed by the publisher.
